# Electronic Sensing Platform (ESP) Based on Open-Gate Junction Field-Effect Transistor (OG-JFET) for Life Science Applications: Design, Modeling and Experimental Results

**DOI:** 10.3390/s21227491

**Published:** 2021-11-11

**Authors:** Abbas Panahi, Deniz Sadighbayan, Ebrahim Ghafar-Zadeh

**Affiliations:** 1 Biologically Inspired Sensors and Actuators (BioSA), Lassonde School of Engineering, York University, Toronto, ON M3J1P3, Canada; panahiyu@yorku.ca; 2 Department of Electrical Engineering and Computer Science, Lassonde School of Engineering, York University, Toronto, ON M3J1P3, Canada; 3 Department of Biology, Faculty of Science, York University, Toronto, ON M3J1P3, Canada; denizsdg@yorku.ca

**Keywords:** field-effect transistor (FET), biosensor, DNA sensor, oral neutrophil, ISFET, BioFET

## Abstract

This paper presents a new field-effect sensor called open-gate junction gate field-effect transistor (OG-JFET) for biosensing applications. The OG-JFET consists of a p-type channel on top of an n-type layer in which the p-type serves as the sensing conductive layer between two ohmic contacted sources and drain electrodes. The structure is novel as it is based on a junction field-effect transistor with a subtle difference in that the top gate (n-type contact) has been removed to open the space for introducing the biomaterial and solution. The channel can be controlled through a back gate, enabling the sensor’s operation without a bulky electrode inside the solution. In this research, in order to demonstrate the sensor’s functionality for chemical and biosensing, we tested OG-JFET with varying pH solutions, cell adhesion (human oral neutrophils), human exhalation, and DNA molecules. Moreover, the sensor was simulated with COMSOL Multiphysics to gain insight into the sensor operation and its ion-sensitive capability. The complete simulation procedures and the physics of pH modeling is presented here, being numerically solved in COMSOL Multiphysics software. The outcome of the current study puts forward OG-JFET as a new platform for biosensing applications.

## 1. Introduction

Field-effect transistor-based biosensors have received significant attention during the last decade due to their unique sensing characteristics, making them valuable sensing platforms for a range of applications such as diagnostics [1,2,3,4,5,6,7,8], gas sensors [9,10], food safety, and environmental monitoring [11]. The most appealing features of FET sensors are their low cost, label independence, and rapid detection of bio-analytes. Label-free detection significantly reduces the sensor production cost and enables a more straightforward detection mechanism for the development of point-of-care devices. FET-based biosensors have shown the fast and low-sample-input detection of various driven diseases, establishing a new avenue in biosensing research [12,13]. In particular, with a demonstrated low limit of detection (LOD), this extraordinary feature has motivated many researchers to develop FET sensors for integration with lab-on-a-chip devices to realize low-cost, hand-held personalized medical diagnostic devices. More importantly, FET-based sensors offer higher sensitivity, mass production capabilities (e.g., CMOS-based ISFETs), and are seamlessly miniaturized for high-throughput integration with lab-on-a-chip technologies. Furthermore, FET-based sensors enable sample charge detection, which allows for the obtaining of rich quantitative biological data from biomolecules in solution such as conformational changes, biomolecule structural variations, and monitoring extracellular potentials in a label-free fashion that is not easily accessible with other sensing mechanisms [2]. On the basis of these characteristics, FET biosensors have been considered as promising candidates in the label-free biosensing area [14,15,16,17,18,19,20].

Thus far, various structures for FET sensor have been introduced that, along with a myriad of biological targets for detection, have established the foundation of many biological FET (BioFETs) sensors. Amongst different BioFETs, the silicon-based BioFETs have witnessed significant progresses in CMOS technology. The unmodified CMOS technology in particular has leveraged silicon-based ISFETs to a commercial grade [21,22]. Developing ISFETs based on the CMOS technology enables significant improvement in scalability with low power consumption, which is extremely important for developing remote biosensing devices. As a result, different structural variants have emerged on the basis of the CMOS fabrication technology, such as the extended gate, the floating gate, and the floating-control gate. The ISFET was created by removing the metal gate on top of the conventional MOSFET to create an exposure to solution by making a solution oxide semiconductor structure [23]. Other forms of BioFETs include incorporation of novel materials as the conductive channel (sensing element) such as graphene, carbon nanotubes, MoS_2_, and metal–organic frameworks (MOF) [24], either in the back gate or solution gate mode [25]. While these materials have shown great potential for different biosensing applications, their scalable fabrication is still challenging.

In terms of integration with lab-on-a-chip for high-throughput fabrication, new BioFET sensor designs are needed to work without the bulky electrode due to its high demand for personalized medical devices for blood or other human fluid testing. Removing bulky reference electrodes enables easier integration with lab-on-a-chip devices since the reference electrode seriously hinders high-throughput packaging [26]. Many attempts have been made to miniaturize the bulky reference electrode (e.g., Ag/AgCl); nevertheless, the downscaling of the materials has caused more drift and unwanted noise issues along with the electrode’s material instability [27]. A practical solution for BioFETs is to develop back-gated structures to use the stable gate as the reference voltage. Therefore, introducing new FET structures based on a back gate is highly desirable for biomedical applications of FET sensors [28,29].

In this paper, we introduce a novel back gate FET sensor called OG-JFET, showing the capability of the sensor operation without a reference electrode in the solution, which makes the sensor integration with lab-on-a-chip more straightforward. The current JFET-based back gate structure has not been introduced for biosensing applications thus far. Furthermore, the OG-JFET requires very low voltage (<0.5 Volt) for the gate, which yields a low-power electronic platform for hand-held point-of-care devices. To better understand the device, we simulated the sensor with COMSOL Multiphysics, along with a detailed discussion on the biasing conditions. The OG-JFET can be considered a p-type channel JFET, with an n-type gate where the other gate has been removed to open the space for introducing chemical/biological samples. Contrary to the previous silicon-based structure that used the bulk oxide as the back gate, in OG-JFET, the channel is controlled through a p–n junction in reverse bias. Previously, the same technology was used for photovoltage field-effect transistor applications, which showed a novel structure for pushing the infrared detection limits in silicon [30] by taking the superior advantage of JFET in signal amplification and back gated design.

The FET sensors have been used frequently for pH sensing applications as they provide miniaturized sensors with high sensitivity that can be integrated with lab-on-chip devices for point-of-care device development [2,31,32,33]. Here, we demonstrated the functionality of the OG-JFET for sensing the ion concentration variation in the solution with pH experiments. The sensor was tested with a phosphate buffer solution with different pH values showing the capability of the sensor to detect the H+ and OH- concentrations in the base solution. The response of pH sensitivity of OG-JFET is also verified with COMSOL simulation to prove its ion-sensitive capability. There are various emerging structures for pH sensing such as extended gate [34] and Fin-FETs [35], as well as the graphene-based FETs [36], which are promising new structures for enhancing the performance of BioFETs.

The DNA molecule is a biomolecule that is extremely important for all aspects of life science [37]. FET sensors have been recognized as sensitive charge detectors for analyzing DNA molecules, providing quantitative data of hybridization and structural conformations [38,39]. Many efforts have been made to use FET sensors for DNA detection [40,41,42]; however, DNA charge analysis has not been reported in dry conditions, which are very important for DNA-based data storage applications. To demonstrate sensor response to a known charged biomolecule, we used ssDNA molecules in different concentrations as a negative charge model, showing increase in the sensor drain-source current when biomolecules are loaded on a sensor. It was demonstrated that sensors can be used to monitor dried ssDNA, which can also be used in DNA sensors. The ssDNA in dry condition was tested to illustrate the possible sensor application in DNA-based data storage, which requires monitoring the mass DNA in a specific spot [43,44,45].

FET sensors have been widely used in cell analysis, showing great potential for cell analysis for different applications [46,47]. The sensor was tested with oral neutrophil cells in ultrapure water to further demonstrate the sensor’s applicability in the direct detection of bioparticles. The ultrapure water droplet containing oral neutrophil cells caused sedimentation of cells on the sensing area, leading to direct cell adhesion on the sensing gate. The existence of cells on the sensing site caused a local variation in the surface charge of the sensor, leading to a change in the source-drain current. In this regard, the sensor demonstrated potential applications in cell sensing and direct bioparticle detection. The negative charge of cells changes the sensor response in the same way as DNA molecules, leading to an increment in output current [48,49].

Knowing that FET sensors have been used for humidity tests [50,51], we tested the sensor in this study with direct human exhalation to show the effect of natural human breath on the response as the humidity model. The majority of human exhalation consists of water vapor that, when it meets solid surfaces, rapidly evaporates. The process has been tested with this sensor, showing the usefulness of the sensor human exhalation analysis.

The device’s functionality for sensing charge on the surface was verified with pH solution, DNA molecule, oral neutrophil cells, and human exhale condensation and evaporation. The models were used to show the operation of the sensor that put the sensor for any charge detection applications from biomolecules to human breath analysis.

## 2. OG-JFET Mechanism of Working

### 2.1. OG-JFET Sensing Principle

**Figure 1:**
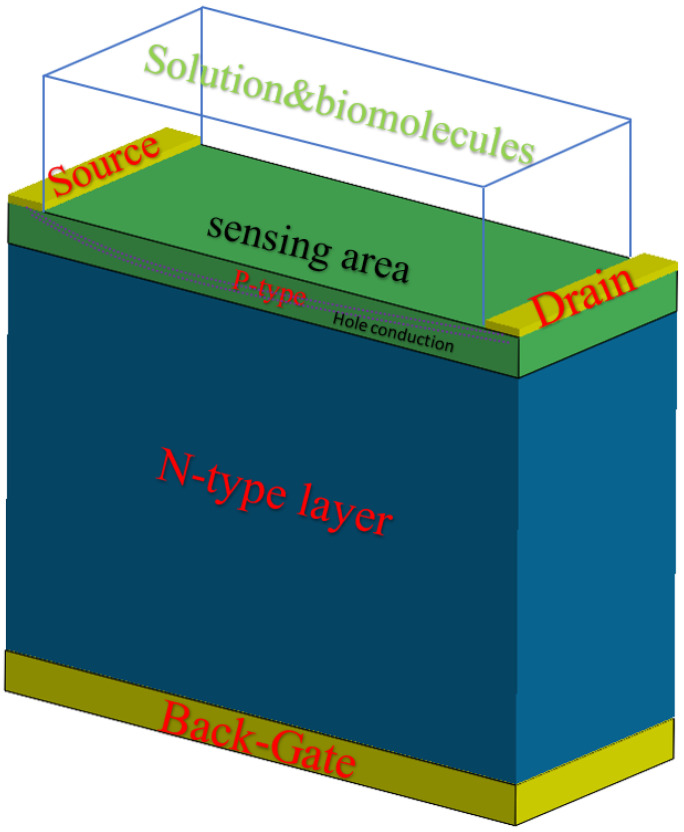
Schematic of OG-FET sensor showing the main material composition of the sensor. The sensing layer of OG-JFET is the p-type silicon between two electrodes of source and drain. The open-gate area is covered with the native oxide layer that usually appears when silicon is exposed to air.

The OG-JFET sensor is fabricated on the basis of a VLSI microfabrication method, which realizes a vertical p–n junction in which the p-type layer is the sensing layer. On top of the p-type layer, two electrodes are deposited, the source and drain, creating a conductive channel between two separate electrodes. The p-type layer and electrodes are epitaxially grown on top of a thick n-type layer connected to a back gate on the other side of the chip. There are holes in the p-type layer as the primary charge carrier that flows from source to drain. The charge transport in the semiconductor sense induces electric field effects from the opened gate area (see Figure 1); consequently, the current in the channel changes when exposed to biomaterial in the opened space. The sensor is similar to conventional JFETs, with the subtle difference that one of the top gates (here n-type) is removed. The sensor’s electrical output curves are expected to be like JFETs, as this is proved in other variants of the JFET for different applications. A thin layer of p-type silicon (1.6 µm) is epitaxially grown on a 450 µm thick n-type silicon to form the OG-JFET main structure. The p-type layer is boron-doped with the density of 5 × 10^15^/cm^3^, and for n-type, it is 1 × 10^18^/cm^3^ with doping of phosphor. A high doped region is created on the backside of the sensor (on the back of the n-type layer), and then through an ohmic contact, the back gate is formed. When a solution or a biometrical meets the silicon in the opened gate area, the charges induce an electric field that influences the positively charged holes. The silicon is not covered with silicon dioxide, but it is a naturally formed oxide called native oxide that is very thin. The silicon dioxide is the first sensing layer of the sensor in contact with the substances and chemical solutions in the opened gate area. Through the Si–OH bonds and ion complexation on the surface of silicon dioxide, we can sense the chemical potential in the solution in the oxide layer that leads to changes in the conduction of holes.

### 2.2. Design and Fabrication of OG-JFET

**Figure 2:**
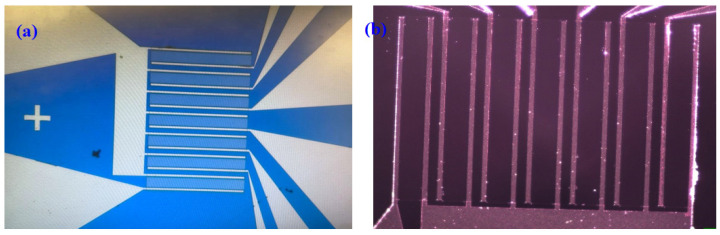
Microscopic images of the designed sensor. (**a**) The image was taken from the prob-station camera, showing the designed sensor with channels and the common source. (**b**) Close-up image under the optical microscope.

**Figure 3:**
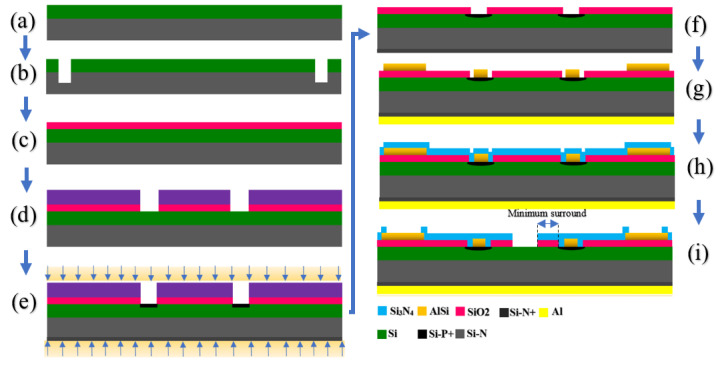
Schematic of OG-FET sensor showing the main material composition of the sensor. (**a**) Substrate wafer (silicon <100>, 100 mm diameter, 420 +/− 25 µm thick, resistivity 0.006 to 0.02 Ohm-cm). (**b**) Alignment marker definition (first mask). (**c**) PECVD SiO_2_ deposition. (**d**) SiO_2_ etching to Si layer (second mask). (**e**) Front- and back-side ion implantation. (**f**) Implantation annealing. (**g**) Front- and back-side metallization and annealing. (**h**) PECVD Si_3_N_4_ deposition (passivation layer). (**i**) Pad and channel opening.

OG-JFET was fabricated through a standard microfabrication process offered by CMC Microsystem (Montreal, Canada). The layout design was performed in L-Edit software and then submitted for fabrication. The sensor was designed on the basis of the common source principle enabling a large pad to connect all sources. The drains were linked to their corresponding large pads. The channel size was 100 µm × 1000 µm (W × L) with a thickness of 1.6 µm. In this design, we had seven channels. The distance between two channels (source to drain) was 40 µm. The width of the source and drain was 20 µm, and their length was 1000 µm. All sources were connected, and the drains were regulated with a switch implemented in the printed circuit board (PCB). The sensor was designed to position a total sensing area of 1000 × 1500 µm^2^ at the center of the chip, as demonstrated in Figure 2. The pads area was 1700 × 1700 µm^2^ with a pitch of 2 mm. The minimum surround for the sensors (the SiO_2_/Si_3_N_4_ layer on both sides of opened channel, see Figure 3 step (i)) was considered at 10 µm. The total sensing area that was exposed to the solution in the current design was 0.7 mm^2^. The schematic of the geometry of the designed sensor is shown in Figure 2.

### 2.3. Fabrication

The sensor fabrication was conducted through a standard microfabrication process, which is shown in Figure 3. The method included seven primary developing processes, as shown in Figure 3. Through the process, first, a substrate wafer (silicon <100>, 100 mm diameter, 420 +/− 25 µm thick, resistivity 0.006 to 0.02 Ohm.cm) is prepared, and then the first mask is applied to define the positions for alignment marker. Subsequently, the PECVD process is performed for SiO_2_ deposition, which is followed by SiO_2_ etching to Si layer that is the opened gate area (shown in Figure 3). The next step is front- and back-side ion implantation, which is followed by implantation annealing. After this stage, the metalization process is initiated, including front and backside metallization and annealing. A thick layer of Si_3_N_4_ is deposited with PECVD to passivate the source and drain. The next step is to open the dielectric layers to make them ready for solution interaction with the silicon channel. The layout of the sensor was designed in L-Edit software. Then, the files were submitted for fabrication at Institut interdisciplinaire d’innovation technologique (3IT), Université de Sherbrooke.

## 3. Methods and Experiments

In this section, the methodologies and details of each experiment are explained. The preparation of PBS (1X) solution with different pH levels, ssDNA solution with different concentrations, oral neutrophil purification, and exhalation analysis are included. Moreover, the characterization devices and the methods of testing the sensor are explained in detail.

### 3.1. Measurement Setup

**Figure 4:**
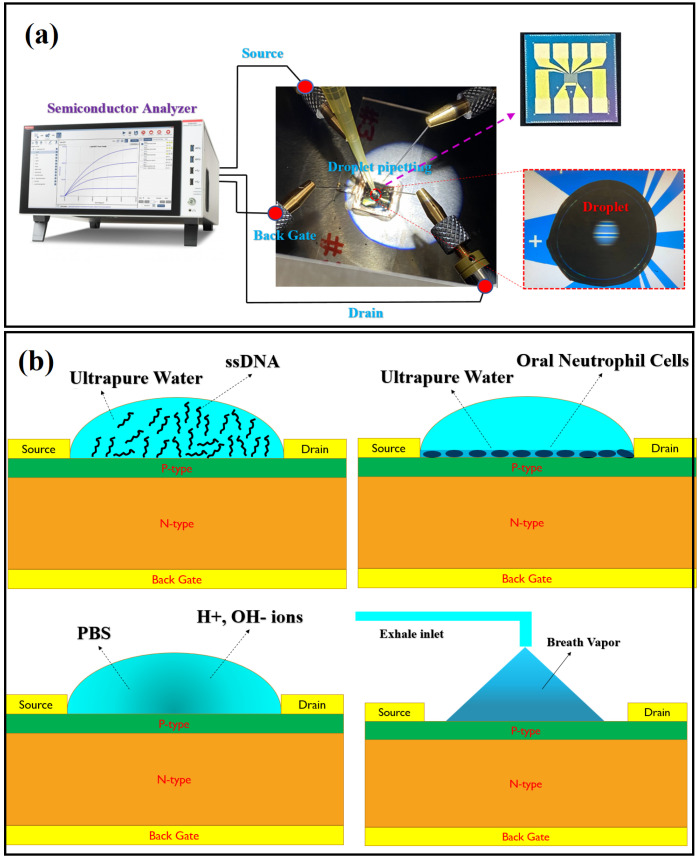
The experiments were set up using probstation and the semiconductor analyzer. (**a**) The probstation was connected to the semiconductors through SMU cables. The droplet containing the analyte of interest was pipetted on the sensor. The droplet volume was 2 µL. (**b**) Different experiments developed for the characterization of sensor capability toward surface charge variation detection.

The OG-JFET was tested with a probe station (MPI TS200-SE Probe Station) that connects the sensor to a semiconductor analyzer (Keithley 4200A-SCS Parameter Analyzer) device, as shown in Figure 4. 

The probstation was connected to the sensor, as shown in Figure 4. The sensor was placed on a glass slide on the conductive copper tape to create a back gate connection. The back gate connection was soldered. To characterize the sensor, we swept the drain voltage between 0 and 10 with a negative voltage, and then the back gate was changed from 0 to 1 in steps of 0.1 V. The back gate voltage was positive to assure the operation of the sensor in reverse bias condition. However, in different experiments, the range of voltage varied on the basis of the condition of the experiment, but the same biasing scheme was preserved.

### 3.2. pH and DNA Sample Preparation

The functionality of the sensor was verified with three samples: the PBS (phosphate buffer solution) with different pH, dried ssDNA, and oral neutrophil cells as the models for showing the sensor response to varying surface charges. The PBS was used as the solution, and different HCl and NaOH solutions were added to the PBS with pH of 7.4 to create different pH solutions. The pH was measured with a pH meter. The DNA concentrations were prepared in ultrapure water solution. The solvated ssDNA molecules in ultrapure water were put on the sensor with pipetting (see Figure 4) and left to evaporate at room temperature. After evaporation of the droplet, the dried ssDNA concentration on the surface of sensor was tested.

### 3.3. Oral Neutrophil Preparation

To prepare neutrophil samples, we advised participants to consume nothing but water within 30 min before sample collection and to then rinse their mouths with water for 30 s. Next, they were asked to wait for 2 min before their oral cavity was rinsed with 10 mL of saline measured using the 15 mL falcon tube and the sample was collected into the 50 mL falcon tube. After one wash, there was a 2 min wait time, and the same process was repeated five times. Then, the final sample was filtered with 2 different sizes of filters. It was distributed into two new 50 mL falcon tubes (25 mL in each, T1 and T2) via 40 μm filters that separated debris and cells larger than 40 μm. After this step, two 11 μm filters that mainly separate epithelial cells (with 20 mL syringes, 11 um white membranes) were prepared and tightened gently into the syringes. T1 and T2 were poured gently and periodically through the membranes. The process was repeated with four 15 mL falcon tubes of 1-time-filtered oral neutrophils through new syringes so that the PMNs were double-filtered. The tubes were centrifuged at 4 °C for 5 min with 2600 RPM, and the supernatant was discarded until 1 mL of the sample remained. Finally, all four samples were collected into one tube and centrifuged for 5 min at 2600 RPM and 4 °C in a microcentrifuge. To dilute the neutrophil sample, we used ultrapure water, and four different concentrations were prepared such that 100% of the first, 75% of the second, 50% of the third, and 25% of the fourth solution consisted of cells. Then each sample was cast on the sensor using a sampler.

### 3.4. DNA Sample Preparation

For DNA experiments, ssDNA (Sigma-Aldrich Company, Burlington, MA, USA) a concentration of 10.0 ± 1.0 mg ssDNA in 1 mL in ultra-pure water (Sigma-Aldrich) was prepared. The prepared solution was then diluted to obtain 101 ng/µL, 180 ng/µL, 270 ng/µL, 301 ng/µL, and 370 ng/µL concentrations of ssDNA. The prepared ssDNA was put on the sensor with pipetting as shown in Figure 4.

## 4. Numerical Simulation of OG-JFET

**Figure 5:**
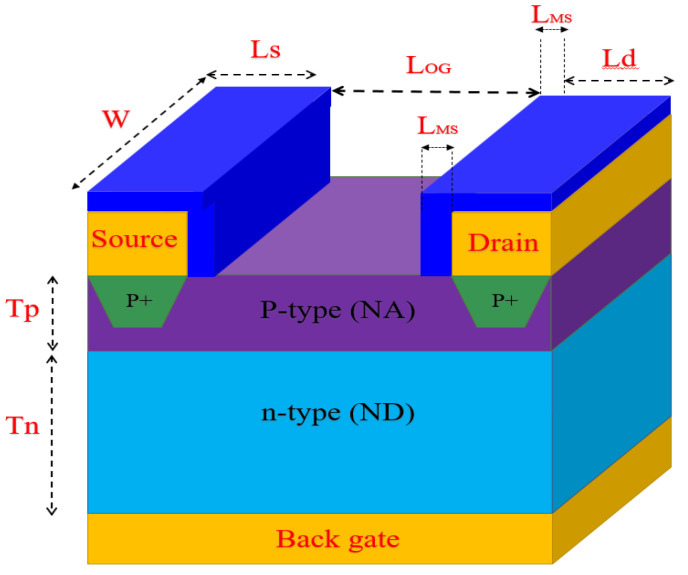
Schematic of OG-FET sensor showing the main material composition of the sensor. tn >> tp and tp >> n++ in terms of thickness. Blue color is the SiO_2_/Si_3_N_4_ layer shown in the fabrication process (see Figure 3) that is used for passivation.

The OG-JFET sensor was simulated in COMSOL Multiphysics software to obtain the transfer characteristic curves. The I-V curves and other physical information that COMSOL simulation provides enable us to better understand of the sensor operation. The *semiconductor module* of COMSOL software was used for the finite element simulation study. To simulate the sensor, we created a 2-D model of the sensor in the model builder of COMSOL according to the size and dimensions shown in Figure 5 that are further explained in Table 1. There were many errors during all the development stages (see Figure 5). The errors in fabrication associated with developing the open-gate area and the wafer itself, as well as the doping-level estimations, were the primary sources of errors in the simulation. Therefore, the simulation is helpful in estimating the fabrication errors to understand the noises associated with the possible fabrication errors.

The principal parameter values used in the simulation are presented in Table 1. However, these values were not perfectly met during the fabrication due to inherent fabrication errors. Moreover, the doping concentration and distributions in both n-type and p-type layers were not precisely preserved. The p-type layer was verified to have a resistivity of 3.0 Ohm.cm +/− 10% as an epitaxial layer on the n-type layer as the substrate with a resistivity of 0.006 to 0.02 Ohm.cm. The n-type thickness was considered at approximately 450 µm in the simulations.

### 4.1. Semiconductor Simulation

The semiconductor module of COMSOL was used to simulate the OG-JFET sensor. It consists of interfaces and integrated models that can solve the non-linear equations of semiconductor devices. COMSOL software solves the semiconductor equations because the energy bands are parabolic and the same temperature for carrier and lattices. In this module, the proposed 2D geometry was meshed for numerical analysis. The following Equations (1)–(5) were solved numerically on the basis of the applied boundary conditions in the semiconductor module of COMSOL software. Maxwell’s equations, Boltzmann transport theory, and Neumann boundary conditions were employed to simulate the sensor. The basic equations used are

(1) $$\rho +=q\left(p-n+N_{d}{}^{+}-N_{a}{}^{-}\right)$$

(2) $$\nabla .J_{n}=-\mathrm{qUn}\;,\;\nabla .J_{p}=-\mathrm{qUp}\;$$

(3) $$J_{n}={\mathrm{qn}\mu }_{n}\nabla E_{c}+\mu_{n}K_{B}\mathrm{TG}\left(\frac{n}{N_{c}}\right)\nabla n+{\mathrm{qnD}}_{n,\mathrm{th}}\nabla \ln\left(T\right)$$

(4) $$J_{n}={\mathrm{qn}\mu }_{p}\nabla E_{v}-\mu_{p}K_{B}\mathrm{TG}\left(\frac{p}{N_{v}}\right)\nabla p-{\mathrm{qpD}}_{p,\mathrm{th}}\nabla \ln\left(T\right)$$

(5) $$E_{c}=-\left(V+\chi_{0}\right)-\alpha \Delta E_{g}\;,E_{v}=-\left(V+\chi_{0}+E_{g,0}\right)+\left(1-\alpha \right)\Delta E_{g}$$

where ψ equals electrostatic potential; q indicates the elementary charge; and n and p are the electron and hole concentrations, respectively. N represents the fixed charge associated with ionized donors. In the above equations, q is the elementary charge; ε means the permittivity of the material; V is the electric potential; ND+ and NA- are ionized donor and acceptor concentrations, respectively; µn is electron mobility; µp is hole mobility; Jn is electron current density; Jp is hole current density; D is diffusion; Eg is bandgap energy; K_B_ is Boltzmann constant; T is temperature; and p and n are the hole and electron concentrations, respectively. Un and Up are the net electron and hole recombination rates for all generation and recombination mechanisms. Fermi–Dirac statistics were also used to solve the probability of carriers’ occupancy in existing energy levels when the system was in its thermal equilibrium. We also tested Maxwell–Boltzmann statistics, but the results were not satisfactory compared to Fermi–Dirac statistics. Here, both p-type and n-type channels were modelled as analytical doping with a Gaussian decay profile.

The Shockley–Read–Hall model for trap-assisted recombination was used to simulate the recombination process. The recombination process involves the trapping of an electron or hole in a defect in the material, and then they are emitted into the valence or conduction band that reduces the number of available carriers. The energy involved in this process is released as heat.

### 4.2. Boundry Conditions and Meshing of Semiconductor Simulation

The boundary conditions were applied on the basis of the OG-JFET physical structure, as presented in Figure 5. The sensors consisted of three ohmic contacts in the source, drain, and back gate regions. The opened gate area and the minimum surrounds were modelled as the insulated surfaces, meaning no flux in/out of this face. This face was opened to air conditions; however, it was not considered for device simulation. The sensor included a p-n junction; thus, the continuity/heterojunction model was used at the junction interface, allowing continued quasi-fermi levels. All surrounding sides of the sensor were modelled as insulation, the same as the opened gate area. All the simulations were performed on the basis of these assumptions of boundary conditions. The p-type channel was meshed with structured rectangular meshes, followed by the unstructured mesh for the n-type channel.

### 4.3. Simulation of Electrolyte Solution

The OG-JFET sensing surface is made of native oxide, which forms naturally when the silicon is exposed to the environment. When this sensing membrane is exposed to the electrolyte solution, the corresponding surface reactions (ion complexations and water molecules) lead to the creation of surface charges, a thin layer of charge amount that depends on the ionic strength and H+ concentration inside the solution (i.e., pH). The formed stacked layer of charges near-surface can potentially influence the surface charge density at the silicon channel in OG-JFET. Consequently, the change in pH can be modelled as interface charge variation at the opened-gate area (see Figure 5), translated as the gate’s effective potential. The cascade effect of solution ions and surface charges are frequently modelled as the source voltage depending on the pH of the solution that can be mathematically demonstrated as Equation (6) [51,52,53].

(6) $$\Psi_{0}=-\log_{e}10\times {\;\delta }_{w}\times \frac{K_{B}T}{q}\times \left(\mathrm{pH}-{\mathrm{pH}}_{\mathrm{pzc}}\right)$$

In Equation (6), pH_pzc_ is the point of zero charges, q represents the charge of an electron, K_B_ is the Boltzman’s constant, T is the temperature in Kelvin, and $\delta_{w}$ is $\frac{\beta }{\beta +1}$ where the β is the sensitivity parameter, i.e., $\beta =q^{2}N_{s}\frac{\eta }{C_{\mathrm{eq}}\times K_{B}T}$. In the sensitivity parameter, N_s_ is the surface site available of the sensing layer (here it is SiO_2_) that is the property of a material (surface effect) when it is in contact with a solution [41]. For SiO_2_, this N_s_ is equal to 5 × 10^12^/cm^2^ [53]. We can also define the η as $2\times 10^{-\left(\frac{\Delta \mathrm{pK}}{2}\right)}$, in which the $\Delta \mathrm{pH}=\Delta {\mathrm{pK}}_{b}-\Delta {\mathrm{pK}}_{a}$.

As the surface is SiO_2_ (native oxide), according to the site-bonding theory, the electrolyte and oxide interaction complex can be represented as the following surface reactions. The sites for bonding oxide and the hydroxyl group can be considered S and OH in the presented responses [54]. The protonation (S-OH^2+^) and deprotonation (SO^−^) species from S–OH occurs due to the adsorption of ions by hydroxyl groups on the sensing layer (here, SiO_2_). The following equations (7,8) describe the phenomena:

(7) $$\left[S-{\mathrm{OH}}_{2}{}^{+}\right]⇌\left[S-\mathrm{OH}\right]+\left[H_{s}{}^{+}\right]$$

(8) $$\left[S-\mathrm{OH}\right]⇌\left[S-O^{-}\right]+\left[H_{s}{}^{+}\right]$$

On the surface of OG-JFET, when the medium is more acidic, the [H_s_^+^] is greater; consequently, the neutral species S–OH groups will accept a proton to form acceptor type and form SiOH^+2^, which leads to a positive charge effect on the outer surface of the native SiO_2_ in OG-JFET, leading to an increase in the current. In the more basic solutions, the concentration of [${\mathrm{OH}}^{-}$] increases to a level more than [$H^{+}$], causing S–OH species to release proton and form the donor type $S-O^{-}$ that contributes to a more negative effect on the surface of the p-type silicon, leading to an increase in the current. For the state at which the interface charge density becomes zero, the pH is called zero charges (pH_pzc_), which can be correlated with reaction constants in (7) and (8) with the following equations:

(9) $${\mathrm{pH}}_{\mathrm{pzc}}=\frac{{\mathrm{pK}}_{a}+{\mathrm{pK}}_{b}}{2}$$

(10) $${\mathrm{pK}}_{a}=-\log_{10}\left(K_{a}\right){\;\mathrm{where}\;K}_{a}=\frac{\left[S-\mathrm{OH}\right]+\left[H_{s}{}^{+}\right]}{\left[S-{\mathrm{OH}}_{2}{}^{+}\right]}$$

(11) $${\mathrm{pK}}_{b}=-\log_{10}\left(K_{b}\right){\;\mathrm{where}\;K}_{b}=\frac{\left[S-O^{-}\right]+\left[H_{s}{}^{+}\right]}{\left[S-\mathrm{OH}\right]}$$

In Equations (9)–(11), K_a_ is the dissociation constant for acid, and K_b_ demonstrates the base dissociation constants. Here, we considered the pH_pzc_ = 2, and the pK_a_ and pH_b_ values used for pH determination on SiO_2_ were 6 and −2, respectively [52,53,54]. Moreover, it is essential to calculate the hydrogen ion concentration at the interface of OG-JFET surface with solution (SiO_2_), which is given as Equation (12) [55]:

(12) $$\left[Hs^{+}\right]=10^{-\mathrm{pH}}\times e^{-\left(\frac{q\Psi_{0}}{\mathrm{KT}}\right)}$$

The variations in [Hs^+^] modulate the surface charge density, which is

(13) $$\sigma_{0}=q\left(\left[S-{\mathrm{OH}}_{2}{}^{+}\right]-\left[S-O^{-}\right]\right)$$

By some mathematical efforts, the following equation will result for the calculation of surface charge density:

(14) $$\sigma_{0}={\mathrm{qN}}_{s}\left(\frac{\frac{\left[{\mathrm{Hs}}^{+}\right]}{\mathrm{Ka}}-\frac{\mathrm{Kb}}{\left[{\mathrm{Hs}}^{+}\right]}}{\frac{\left[{\mathrm{Hs}}^{+}\right]}{\mathrm{Ka}}+\frac{\mathrm{Kb}}{\left[{\mathrm{Hs}}^{+}\right]}+1}\right)$$

With the abovementioned formula, we can calculate the equivalent surface potential of each pH value, as was used in other works, and then use it as input in the COMSOL simulation.

## 5. Results

### 5.1. Sensor Functions in Dry Mode 

**Figure 6:**
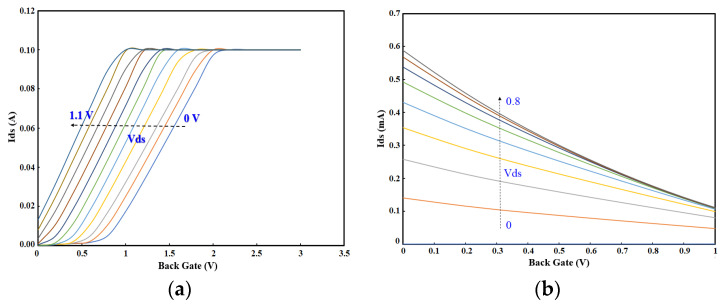

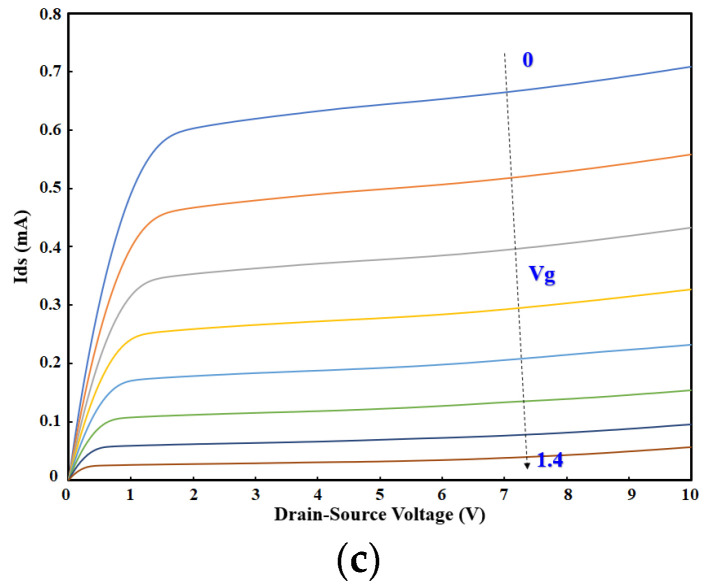
The current-voltage curves of the OG-JFET sensor for two bias conditions, forward and reverse. (**a**) Ids-Vgs of forwarding bias. (**b**) Ids-Vgs of reverse bias condition. (**c**) Ids-Vds curve for reverse bias condition (steps: 0.2 V).

The OG-JFET sensor consists of a vertical p-n junction that enables the control of the channel through the back gate (see Figure 6). According to the structure, the sensor can be operated in two modes, reverse bias and forward bias. The reverse bias guarantees that the p–n junction is turned off; as a result, there is only a current inside the channel (p-type, see Figure 6). However, there is another mode of working for this sensor, the condition of forward-bias, wherein the p–n junction is turned on if the voltage difference between the p-type and n-type junction is more than ≈0.67 V. If the voltage is higher than this value, the p–n junction is opened and the current directly flows from back gate to the drain, which is more current compared to the reverse bias current. The transfer characteristics of the OG-JFET for both states of biases are shown in Figure 6.

According to Figure 6, the I-V curves for the forward bias condition demonstrate the operation of the sensor in elevated current when the p–n diode is turned on. The experiments are conducted at room temperature. According to the data, the p–n junction will be turned on at a voltage of 0.67 V for a p-type of concentration of 5 × 10^15^ and n-type concentration of 1 × 10^18^, meaning that when the Vd-Vg is equal to or higher than this value, the current will directly flow from the back gate to the drain. However, the reverse bias condition works with the current in the p-type channel that is lower than the current in forward bias condition. This is expected as a huge channel with an area of 10 × 10 mm and length of ≈450 µm is in front of the current from the back gate to the drain, in forward bias. The Ids-Vgs for the reverse condition are depicted in Figure 6. The graph shows a linear decay of the current versus the back-gate voltage, as shown in Figure 5. The back gate is used to control the space charge in the channel by which the current can be modulated. When the I-V curves were obtained from the fixture setup, the sensor was in dry condition, and there was no solution or other material on it. The sensor response was repeated multiple times to assure the consistency in response.

The tests were conducted in the fixture setup (the microfluidic is attached). The sensor’s stable function represents the sensor’s good working condition, showing good alignments.

**Figure 7:**
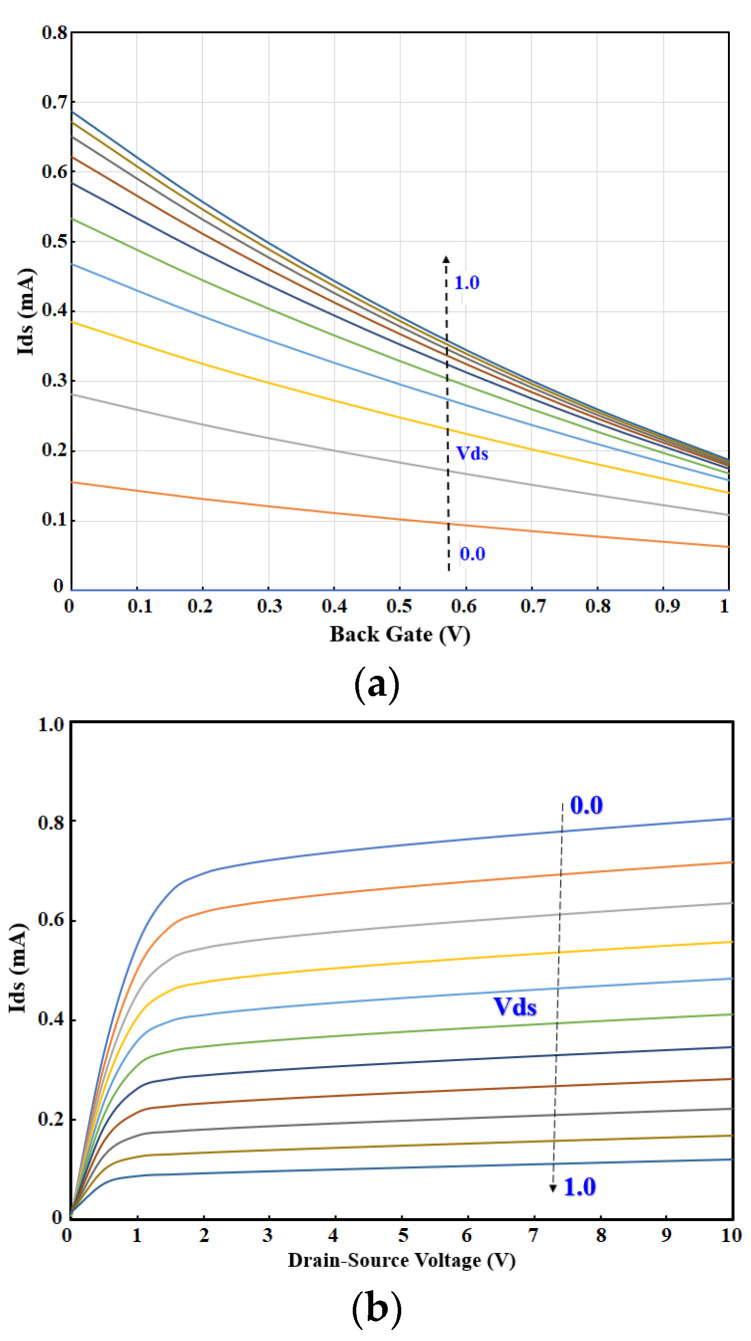
Transfer characteristics of OG-JFET sensor derived from COMSOL simulations. (**a**) Reverse-bias Ids-Vgs. (**b**) Reversed-bias Ids-Vds.

The structure is new; thus, the sensor’s operation needs to be validated with simulation to gain insight into the physics of the sensor. The COMSOL Multiphysics software was used for the semiconductor analysis of OG-JFET. There are inherent non-idealities associated with the fabrication of the sensor itself during the standard fabrication procedures shown in Figure 3, which leads to errors in simulation. However, the user-friendly features of COMSOL software enables multiphysics simulation of the sensor to take care of all physical phenomena on the sensing surface, such as molecular detection and solution simulation. The simulation results (see Figure 7) ideally represent the sensor response trend, which can be used to design the sensor for other geometries before fabrication. 

### 5.2. Sensor Response to Surface Charge Variations

**Figure 8:**
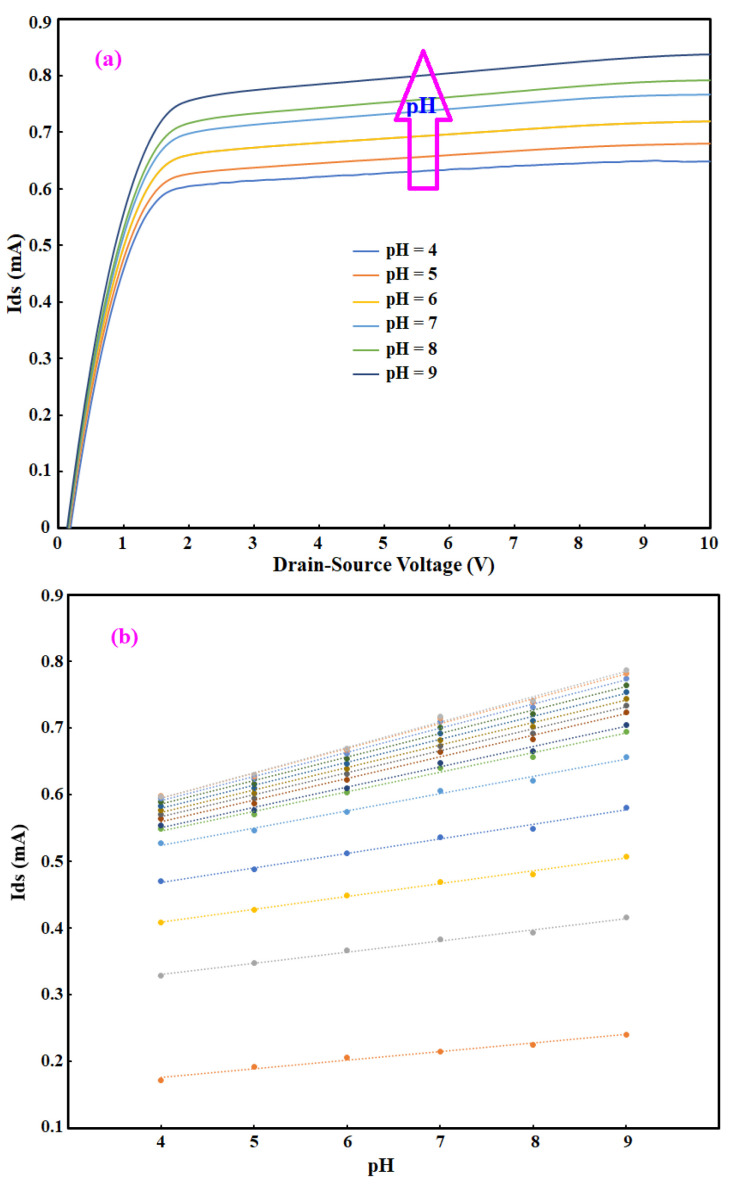
Drain-source voltage graphs for different pH values for OG-JFET at Vg = 0. (**a**) Ids-Vds curve for different pH values. (**b**) The current sensitivty for different values of Vds.

The OG-JFET was tested in the probstation shown in Figure 4 to demonstrate its functionality to detect the surface charge. The sensor was connected to a semiconductor analyzer, and the liquid with a specific load of ssDNA, pH, and oral neutrophil was loaded on the sensor in a droplet form as shown in Figure 4. The fluid was not in continuous mode as it changed the surface charge concentrations due to the motion of fluid in the sensing area; therefore, considerations were taken not to mitigate the droplet liquid condition in the sensing area that creates turbulences inside the charge layers on the sensor. To model the surface charge variations, we used phosphate buffer solution (PBS) as the primary fluid with the extra addition of HCL and NaOH to create different concentrations of ion charge in the solution. With titration of PBS, the solution samples were prepared and then loaded on the sensor with a pipette. After testing a specific solution with a known pH, the sensor was rinsed with DI water to prepare it for the next pH solution. Before every try with pH solution, the sensor response was measured to ensure it returns to the baseline condition. Figure 8 demonstrates the sensor response to pH solution. To measure the baseline, we obtained the current-voltage curves from the same device, and then we determined the difference in the current to assure a returing to the same current in a specific back gate voltage.

Figure 8 demonstrates the sensor response of the sensor when the concentration of hydrogen ions was changed in the microfluidic channel. The variations in the concentration of H+ and OH- were generated by producing different pH solutions. This is an excellent way to characterize the sensor functionality in response to the ion/species concentration in the solution. As Figure 8a shows, the curves for all pH values were distinctively separated in the saturation region, which shows the effectiveness of OG-JFET in detecting the change in ion concentrations. The current sensitivity analysis was performed on the sensor and is shown in Figure 8b. The current sensitivity was measured for different Vds values: 0.5 V, 0.8 V, 1.0 V, 1.5 V, 2.0 V, 3 V, 4 V, 5 V, 6 V, 7 V, 8 V, 9 V, and 10 V, as shown in Figure 7 from the lowest curve to the last one. The current sensitivity for these values of Vds changed from 2.0 × 10^−5^ A/pH at low Vds voltages to 4.0 × 10^−5^ A/pH in higher Vds in the saturation region. Figure 8b shows that in the saturation region, the current sensitivity was almost the same and more significant than the ohmic region (linear part of Ids-Vds). This current sensitivity enhancement dependency on the Vds values should be considered in the design of sensors for specific applications.

**Figure 9:**
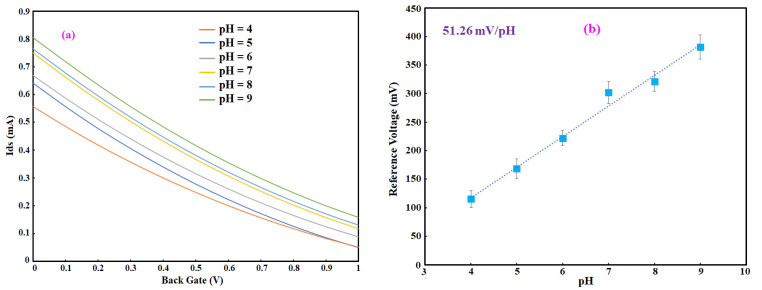
Drain-source voltage graphs for different pH values for OG-JFET. (**a**) Ids-Vgs curve for different pH solutions from pH = 4 to pH = 9. (**b**) The voltage sensitivity relative to the back gate reference voltage.

The existence of a back gate in the structure makes the sensing process more straightforward than when there is a reference electrode in the solution. To show the performance of the sensor in the back-gate structure, we conducted pH tests. The Ids-Vgs variations are plotted in Figure 9, demonstrating the effect of pH variations on the Ids-Vgs (drain-source current versus back gate voltage). As it can be seen, the pH increment enhanced the current in the p-type channel. The sensor was biased in reverse mode, meaning that there was no current in the p–n junction. The sensor showed a sensitivity of 51.26 mV/pH, as shown in Figure 9. To obtain the voltage sensitivity of the device, we plotted the Ids-Vgs; then, the reference current was defined for analysis of voltage sensitivity. As shown in Figure 9, the voltage required to be applied on the back gate to maintain a constant current of 0.5 mA in the main p-type channel was defined as the reference voltage. By plotting that voltage versus the pH values, we were able to calculate the voltage sensitivity, which is the slope of the linear fit.

The sensor (OG-JFET) showed a super-Nernstian pH sensitivity of 51.26 mV/pH, which is more than the limit of this value (<40 mV/pH) for SiO_2_ sensing membrane in previous research works on ISFET [56]. Despite this range of pH sensitivty for ISFETs with SiO_2_, a sensitivty of 47 mV/pH (close to our sensitivty) was reported for silicon nanowire-based ISFET with SiO_2_ sensing membrane, which is an example of sensitivty more than the reported limit for SiO_2_ (≈40 mV/pH) [57]. The linearity of the pH sensitivty is also another impotant point to notice. The reported curves in the litreture are not completely linear for SiO_2_, but there are some cases that have revealed linear response such as [57].

More disucssion on the pH sensitivty is found in Section 6.

### 5.3. Simulation of Surface Charge Effects

**Figure 10:**
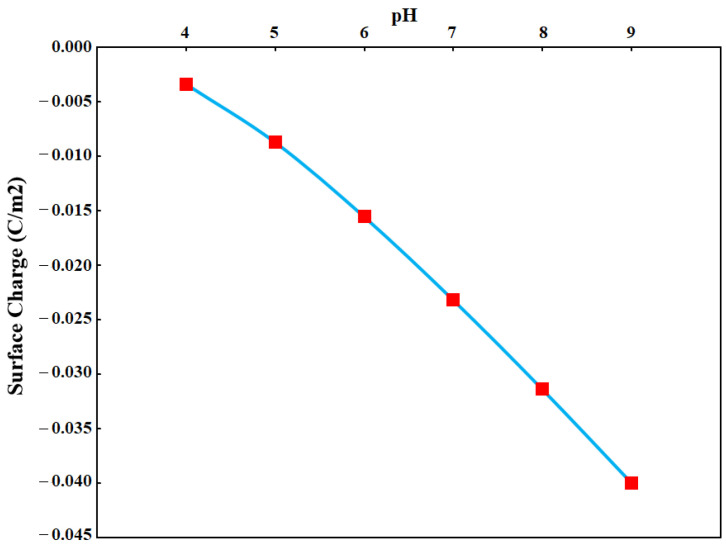
The estimated native oxide of OG-JFET surface charge equivalent to pH.

To better understand the sensor response to pH, change as a model of surface charge modulation, we conducted a simulation in which the pH effects on the sensor drain-source current were simulated. The pH effect was assessed by measuring the effective surface charge due to the existence of ions and solution on the oxide surface on the basis of modelling. The pH equivalent surface charge was calculated and is shown in Figure 10.

The surface charge equivalent to pH was calculated from site bonding modelling. The surface charges were then used in the COMSOL to estimate the OG-JFET sensor response to pH variations. The model allows us to gain insight into the physics of the OG-JFET sensor in response to the surface charge variations. 

**Figure 11:**
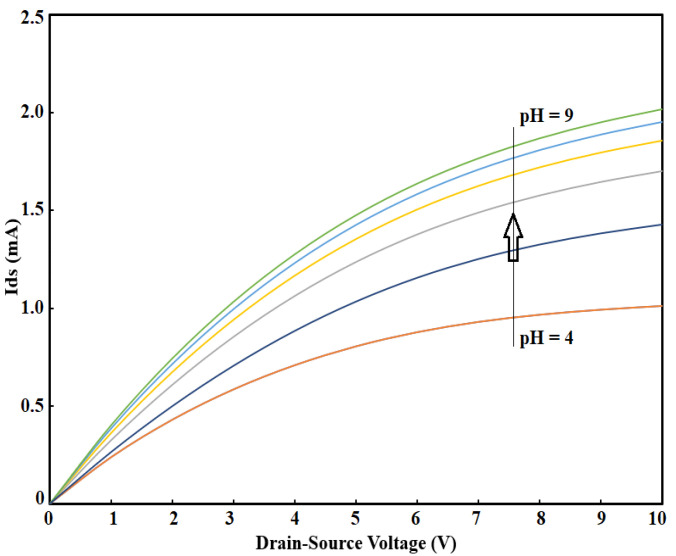
COMSOL simulation results of the pH variations effect on the sensor results.

Figure 11 demonstrates the effect of the pH variations on the OG-JFET. The COMSOL simulation yields the trend of pH variations in agreement with the experiment. According to the experiment, as the pH increases, the surface net charge will become more negative. As a result, the hole conduction in p-type semiconductors will increase due to this effect. The graph was measured in a constant back gate (here, Vg = 0), showing the effectiveness of COMSOL software to estimate the OG-JFET response trend when different surface charges are applied. This implies that COMSOL can potentially be used for multiphysics problems and specific multiscale designs of biosensors. However, the COMSOL simulations come with approximately 11% error, which is mainly due to the non-idealities and errors in the fabrication process and the physical model of pH surface charge that we used to estimate the surface charge values on the oxide region. Another possible source of error might be due to the oxide sites density estimation by the model that is different from the reall concentration in the experiment.

### 5.4. Sensor Response to DNA Concentration

**Figure 12:**
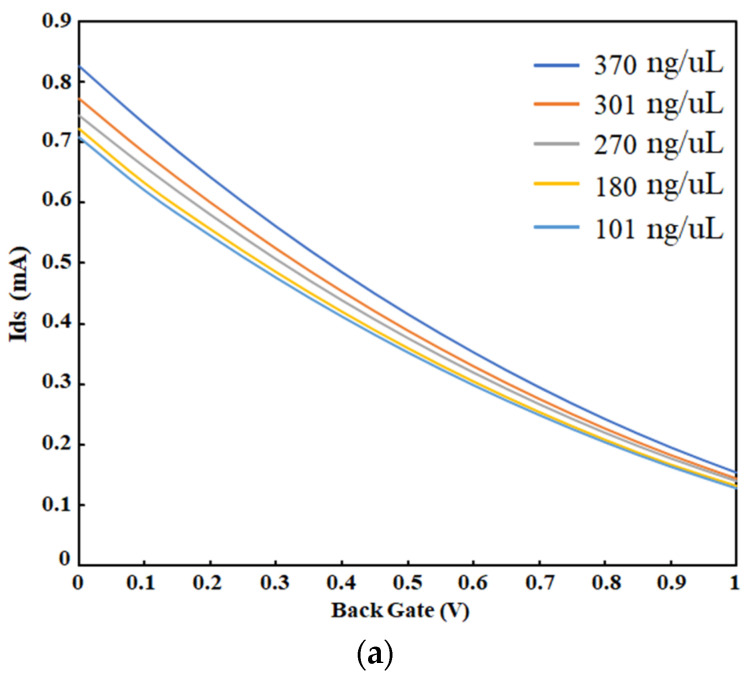

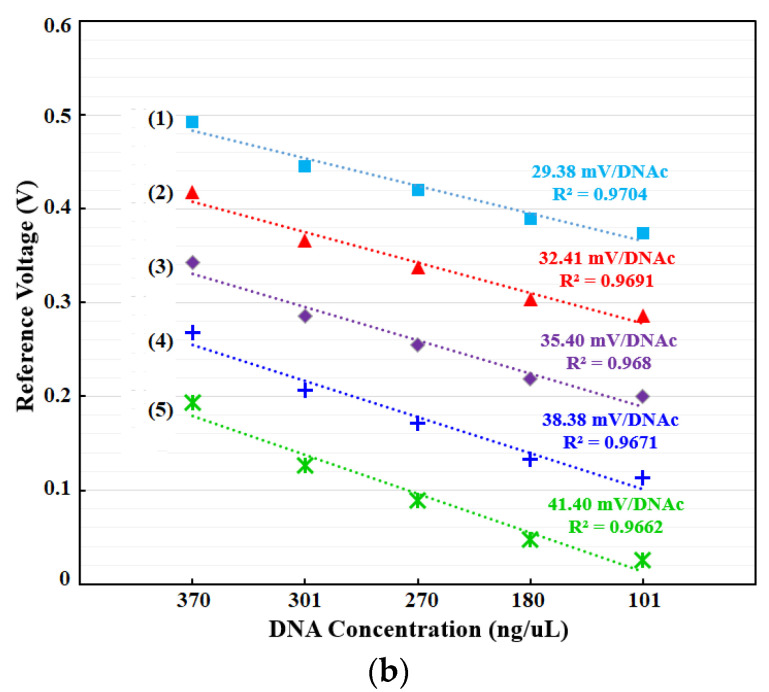
Sensor response to single-strand DNA concentration. (**a**) Ids-Vgs curve for different concentrations of ssDNA (370 ng/uL, 301 ng/uL, 270 ng/uL, 180 ng/uL, 101 ng/uL). (**b**) Voltage sensitivity results of OG-JFET response to ssDNA concentration at different reference currents of (1) 0.45 mA, (2) 0.50 mA, (3) 0.55 mA, (4) 0.60 mA, and (5) 0.65 mA.

Single-strand DNA (ssDNA) molecules were prepared with different concentrations in ultrapure water and tested on the OG-JFET to test sensor response to known charged biomolecules. A droplet of ssDNA solution in ultrapure water was placed on the sensing area, and the sensor drain-source current was monitored. The ssDNA molecules are negatively charged molecules; due to the accumulation of negative charge on the p-type layer, the current was expected to increase in the same way that increasing pH affects the sensor. Negative charge accumulation causes the attraction of holes in the channel, which leads to current enhancement. The Ids-Vgs diagram was recorded in each concentration of ssDNA in a constant Vds of 10 V, as demonstrated in Figure 12. The experiments were conducted 10 times, and the graph shows the averaged response of the sensor in a constant Vds. The experiments were all conducted at room temperature. As the ssDNA molecules were in ultrapure water, it was guaranteed that no charge existed in the solution, and the sensor gate was only affected by the ssDNA molecules’ charge.

Figure 12a demonstrates the Ids-Vgs curves of OG-JFET when dried ssDNA were placed on the sensor. The droplet containing ssDNA was left to evaporate, and after the completion of evaporation, the sensor response was recorded. A 2 µL droplet was placed on top of the sensor, which covered all the sensing areas, as shown in Figure 4. The sensing area was 0.7 mm^2^, which was large enough to accommodate the droplet. Therefore, all the DNA molecules, after drying, were expected to cover the sensing area. As the sensor is sensitive to light, the sensor was placed entirely in darkness in the probstation cage during the experiments. Figure 12b demonstrates the voltage sensitivity curves of the sensor to ssDNA molecules. A sensitivity of 41.40 mV/DNAc was determined on the basis of the characterization. However, the sensitivity was analyzed for other drain current values, which is shown in Figure 11. For drain-source currents of 0.45, 0.50, 0.55, 0.60, and 0.65 mA, the sensitivities were determined to be 29.38, 32.41, 35.40, 38.38, and 41.40 mV/DNAc. The DNAc is the concentration of DNA unit that here was ng/µL.

### 5.5. Direct Human Exhalation Test

**Figure 13:**
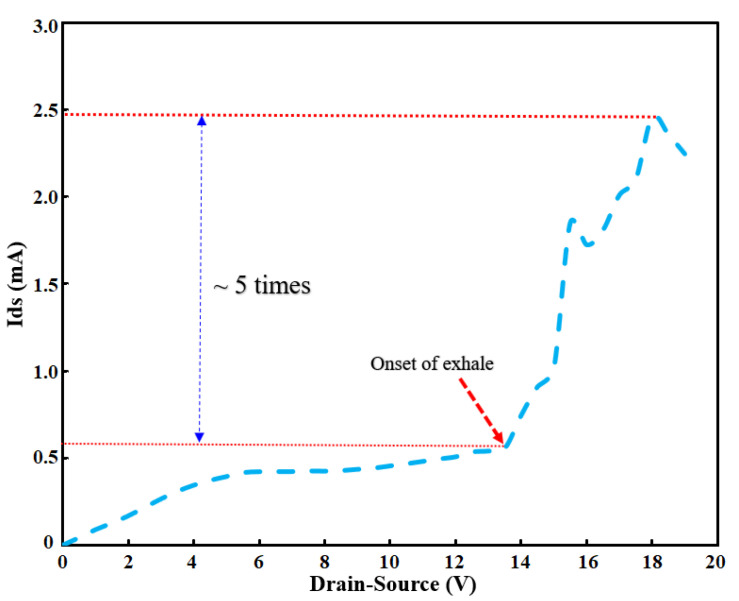
The result of the human exhalation test on the OG-JFET.

To test the effectiveness of OG-JFET in measuring surface wetness upon applying a vapour, we conducted the human exhalation test. As shown in Figure 13, when the sensor sensed the exhalation’s condensation, due to the creation of a very small droplets of water due to the condensation of the vapour, the sensor surface charge changed suddenly. The change from 0.5 to 2.5 mA occurred in <3 s, which shows the significant sensitivity of the sensor towards the sudden surface charge variation. 

A tube was used to direct the exhalation on the sensor that causes the rapid change in the sensor signal (see Figure 4). This simple experiment was conducted to demonstrate the application of this sensor for charge analysis of human exhalation. The ≈5 times increase in drain-source current showed the charge effects on the enhancement of sensor response.

### 5.6. Sensor Response to Oral Neutrophils

**Figure 14:**
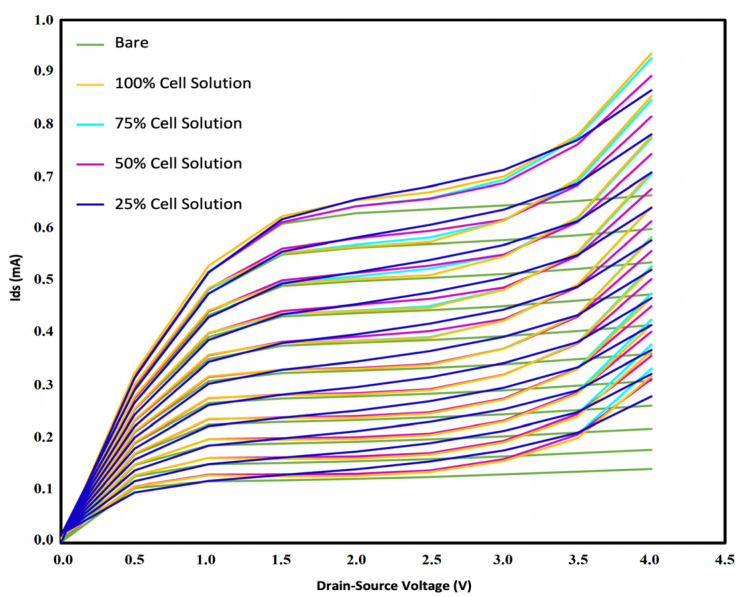
The sensor response to different concentrations of cell solutions in comparison to bare condition.

After the preparation of the cell solutions using ultrapure water, we cast 2 µL of the samples on the sensing region with a sampler. The surface of the sensor was subsequently washed with isopropanol alcohol (IPA) to eliminate the remnants from the previous test. This step is necessary to ensure that the sensor returns to its initial condition before each test. For this purpose, 2 µL of IPA was casted on the sensing region of the sensor and sucked in order to remove the remnants. After repeating this process three times, we left the sensor aside to dry. Next, 2 µL of the second sample was cast on the sensor, and the current of the drain source was recorded. Similar to the increase of pH and ssDNA concentration, the existence of negatively charged neutrophil cells resulted in the accumulation of holes in the channel, which caused an enhancement in the current. Figure 14 represents the sensor response to different concentrations of cell solutions. The curves for each concentration can be differentiated clearly from the others, which confirms the capability of OG-JFET in distinguishing different cell levels. The current sensitivity analysis was performed on the sensor for different Vds values: 1 V, 1.5 V, 2 V, 2.5 V, 3 V, 3.5 V, and 4 V. Cell tests were performed using the back gate of the sensor to examine its proficiency. The variations of Ids were recorded in different Vgs. As can be inferred from Figure 14, the increase of the concentration enhanced the p-type channel’s current. The experiments were all conducted at room temperature. As the cells were in ultrapure water, it was guaranteed that no charge existed in the solution, and the sensor gate was only affected by the neutrophil cells’ charge. 

**Figure 15:**
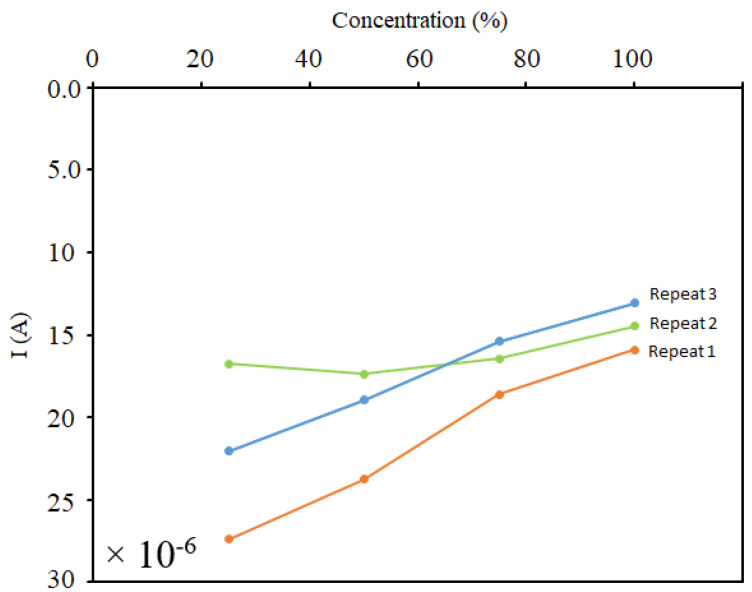
Analysis results of three repeats of testing current variations in different concentrations of neutrophils.

**Figure 16:**
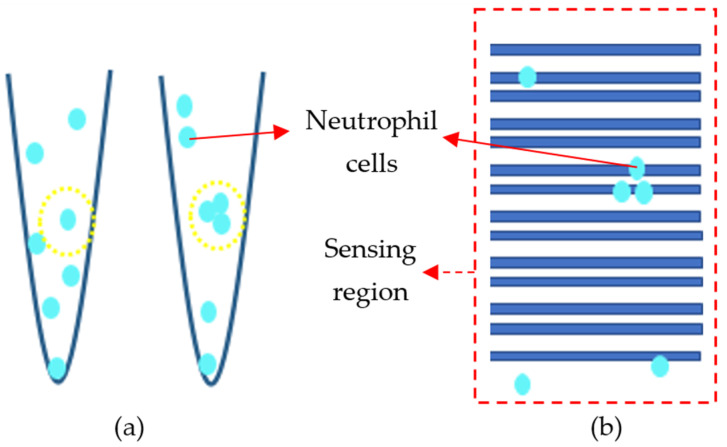
Nonidealities in neutrophil measurement: (**a**) non-uniformity of the cell distribution in the sample, and (**b**) non-uniformity of cells cast on the surface of the sensor.

In order to examine the repeatability of the device, we conducted the test three times. The result of the analysis can be seen in Figure 15. It demonstrates that increasing the concentration of the cells in the solution resulted in the increase of electric current. Almost all three trials followed this trend, which showed that the sensors were responsive, functional, and repeatable. However, its sensitivity could be further improved by decreasing the incidence of some inevitable situations. There were two possible reasons for the low preciseness of the device. The concentration of cells can vary due to the poor uniformity of the cell distribution in the sample. A high rate of precipitation increases the probability of error while sampling and sets the stage for imprecise sample casting. Furthermore, the location of the cells on the sensing region is of high importance since it is not possible to visualize cells on the surface, and therefore we cannot have an exact estimation of their final location (see Figure 16). They might be in a region that cannot be sensed, and as a consequence, the sensitivity decreases. It is noteworthy that neutrophil cells have a size of 9–15 µm, whereas the DNA molecules are between 2.2 and 2.6 nm. This fact results in the low charge density in neutrophils compared to the DNA. It means that a large of these negatively charged cells are needed in order to accumulate the holes in the sensing region of the sensor and trigger a response.

The Debye length plays an important role in the charge detection in BioFET sensors. In the experiment of oral neutrophil cells, the cells were in ultrapure water that had very low ion concentration, causing a higher Debye length for detection of biomolecular charge due to cells’ surface charge. Furthermore, for the ssDNA molecules, sensing is based on the charge effects due to binding of ssDNA with silicon oxide. The oxide charging mechanism is not similar to the solution-based detection in BioFETs in which Debye length plays an important role. The charge of ssDNA in dry mode requires further theoretical and experimental analysis. For the PBS (1X) solution, the Debye length is expected to be between 0.7 and 2.2 nm in biological solution [58]. Here, as we had the native solution dioxide with PBS, we therefore expected that such a range of Debye length exists.

## 6. Discussion

The OG-JFET sensor was fabricated and tested with pH solution, ssDNA molecules in dry mode, oral neutrophil cells, and exhalation moisture to demonstrate its capability towards sensing the surface charge variations. In the pH experiments, it was interesting that the sensor indicated a high sensitivity (super-Nernstian region) of 51.26 mV/pH for the native silicon dioxide sensing layer in the open-gate sensing area. In previously reported works for silicon dioxide, the pH sensitivity was reported to be <40 mV/pH [59,60]. However, the sensitivity of SiO_2_ was reported to be 47 mV/pH in [57], showing a close sensitivity to the OG-JFET pH sensitivity in this work. The native oxide naturally grows on the silicon; however, the environmental condition affects it growth, thickness, and distribution over the period of time. It has been proven that exposure of silicon to air causes native oxide formation with thickness of ≈2 to ≈10 Å for silicon in room temperature [61]. The OG-JFET is exposed to air after fabrication and in the shipping process; therefore, on the basis of the precise analysis of Morita et al. [61], it is expected to have a native oxide layer with certain thickness in the opened-gate area (exposure of silicon to air) of OG-JFET. The study of [61] offers a model for formation of native oxide when silicon is exposed to air, suggesting the existence of oxide sites that set the stage for ion complexation and pH sensitivity as a result [61]. However, the distribution of these available sites on the outermost layer of oxide is not as uniform as a deposited layer of SiO_2_ in other ISFETs that might have affected the sensitivity of sensor. Further studies are required for surface oxide site bond analysis of OG-JFET and its distribution throughout the sensing area for better understanding of surface effects on the comparatively higher sensitivity of OG-JFET.

The sensor was tested with dried ssDNA molecules. The dry ssDNA mass monitoring is of high importance for development of next-generation DNA-based data storage platforms in digital microfluidics [45]. In this study, the ssDNA molecule solution was left to dry on the sensing area. The main mechanisms of ssDNA attachment on the surface of SiO_2_ is the hydrophobic interaction and also phosphate–silanol binding that occurs despite the negativity of ssDNA and negative surface charge of SiO_2_. The ssDNA concentrations were prepared in ultrapure water and cast on the sensor and left to dry. In this condition, the ssDNA created the hydrophobic and silanol–phosphate binding, leading to attachment of ssDNA on the OG-JFET open-gate area. The bonding led to extra negative charge on the very thin layer of oxide that increased the current. While experiments showed the current enhancement due to binding of ssDNA and native SiO_2_, more in-depth theoretical and experiments are required for understanding of surface charge of oxides when dry DNA is placed on its surface, as well as the resultant charge profile of DNA. 

In another part of the study, oral neutrophil cells were cast on the sensor. The cell concentrations were prepared in ultrapure water that assured the existence of oral neutrophil on the sensor. Previous studies have demonstrated the negative overall charge densities on neutrophil cells [48,49]. Neutrophil cells have a negative surface charge that has been stated in some research studies. For example, Marco et al. [49] investigated the effect of changes in the composition of membrane phospholipids on the net cytoplasmic membrane surface charge. They successfully demonstrated that the combination of PS, PIP2, and PIP3 in the membrane resulted in a high negative charge at the plasma membrane of neutrophils. In another study, it was shown that the negative surface charge of the neutrophil cells decreased when incubated with degranulating stimuli. They also reported that the immature neutrophils that are only produced in the bone marrow are highly negative in charge. However, this charge decreases by degranulation and cell maturation [59]. The interaction of surface negatively charged membrane of these cells probably causes increment in the negativity of the surface, contributing to the enhancement in the hole current in the p-type conductive layer of the sensor.

## 7. Conclusions

A new field-effect transistor biosensor is introduced in this work for life science applications from biomolecular to biological cell analysis. The advantage of OG-JFET is that it offers control of the channel conduction through a back gate by which the need for a bulky reference electrode in the solution is eliminated. Moreover, the sensor provides a wide sensing area on top of the sensing channel without an oxide layer, enabling an easier sample introduction and fluidic integration as bulky reference electrodes frequently used in the other in the solution make the integration significantly simpler for developing complex lab-on-a-chip designs as an integrated biosensing element. The sensor is operated in low voltage (<0.5 V) of back gate, which is a low-voltage device for developing remote biosensing applications. Furthermore, the mass production capability promises this technology for a myriad of biosensing applications for the biosensor community. 

To demonstrate the sensor functionality in terms of response to the change in surface charge, we tested the sensor with pH solution, ssDNA molecules, oral cell neutrophils, and human exhalation vapor as a model for solution charge biomolecule, cell detection, and moisture detection, respectively. We calculated a sensitivity of 51.26 mV/pH, 41.4 mV/ssDNAc, 0.15 mA/%-cell for ion solution, dried ssDNA, and oral neutrophil cell detection, respectively. Moreover, we have shown that the sensor was sensitive to the human exhalation-generated moisture with five times increment in the source-drain current upon exposure to the exhalation. 

Furthermore, COMSOL simulation modeling was also provided for multiphysics simulation of OG-JFET to ensure the sensing mechanism of the sensor was working towards surface charge detection. The simulation model validated the correctness of experimental curves. The resultant surface charge of pH solutions was calculated and applied in the COMSOL Multiphysics simulation to estimate the trend of sensor response to the pH solution. The model showed the increment in the response of the p-type channel to increment in pH. However, the introduction can be extended in future works to model more complex surface interactions for multiscale simulation of OG-JFET.

On the basis of the successful experiments of OG-JFET with pH solution, oral neutrophils, and ssDNA biomolecules, we put forward this technology as a new mass-producible BioFET sensor for the biosensing community.

## Figures and Tables

**Table 1:** Design parameters used in simulation and fabrication of OG-JFET.

Design Parameters	Values
P-type length (L_OG_)	100 µm
P-type doping (N_A_)	5 × 10^15^/cm^3^
P-type channel thickness (tp)	1.6 µm
Channel depth (3D, not shown)	1000 µm
N-type length	100 µm
N-type thickness (tn)	450 µm
N-type doping (N_D_)	1 × 10^18^/cm^3^
Source/drain length (Ls, Ld) and width (W)	20 µm and 1000 µm
Source/drain implantation doping (P+)	2 × 10^16^/cm^3^
Source/drain minimum surround (L_MS_)	10 µm
